# Corona-Impfstoffe im Überfluss — was dann?

**DOI:** 10.1007/s10273-021-2895-4

**Published:** 2021-04-17

**Authors:** Dieter Cassel, Volker Ulrich

**Affiliations:** 1 Wirtschaftspolitik und Gesundheitsökonomie, Universität Duisburg-Essen, Lotharstr. 65, 47057 Duisburg, Deutschland; 2 Rechts- und Wirtschaftswissenschaftliche Fakultät, Universität Bayreuth, Universitätsstraße 3, 95447 Bayreuth, Deutschland

**Keywords:** I10, 111, 118

## Abstract

Seit Beginn der Corona-Impfkampagne in Deutschland am 27. Dezember 2020 waren knapp drei Monate später 10,5 Mio. Dosen verimpft. Das waren durchschnittlich 126.000 Dosen pro Tag mit steigender Tendenz: Am 15. März 2021 waren es schon mehr als 250.000 täglich. Damit konnten aber erst 3,2 Mio. Personen vollständig immunisiert werden. Um Herdenimmunität zu erreichen, die wegen der hoch infektiösen Mutanten eine Immunisierungsquote der Bevölkerung von etwa 80 % erfordern dürfte, müssten aber gut 65 Mio. Personen mit 130 Mio. Dosen geimpft sein. Beim jetzigen Impftempo würde es noch bis in den Sommer 2022 dauern, um das Immunitätsziel zu erreichen. Kommt es nach dem Impfstoffmangel bald zu einer Knappheit der Impfkapazitäten? Könnte die Herdenimmunität an mangelnder Impfbereitschaft scheitern?

## Abstract (en)

A total of 3.2 million people in Germany have been fully immunised so far. However, in order to achieve herd immunity, which is likely to require an immunisation rate of around 80 % due to the highly infectious mutants, approximately 65 million people need to be vaccinated. The current “imbalance” in the vaccination campaign is largely due to the limited supply of vaccines. Since J&J’s vaccine has recently been approved by the EU and additional production facilities are available, it is foreseeable that the capacities of the vaccination centres will no longer be sufficient. Moreover, at some point the existing unwillingness to vaccinate could become the final bottleneck in reaching herd immunity. This paper provides some calculations for these relationships. Against this background, it is questionable how well-prepared the present system is to cope with these foreseeable problems.

Das Bundesministerium für Gesundheit (BMG) hatte Ende Oktober 2020 erstmals die Umrisse seiner „Nationalen Impfstrategie Covid-19“ veröffentlicht und daraufhin Anfang November mit der Gesundheitsministerkonferenz (GMK) die Aufgabenteilung zwischen Bund und Ländern als Grundlage für eine bundeseinheitliche Impfkampagne vereinbart. Die dazu erforderliche „Impfpriorisierung“ fehlte aber noch; sie sollte von der Ständigen Impfkommission (STIKO) nachgereicht werden.[1] Auch waren noch keine Impfstoffe zugelassen und neue Impfkapazitäten erst im Aufbau. An eine zeitlich und quantitativ exakte Planung der Impfkampagne war also noch nicht zu denken.

* Die Verfasser danken Dr. Andreas Heigl, München, und Dr. Andreas Jäcker, Weilheim, für ihre pharmaökonomische Expertise.

1 Dies geschah erst mit einem Beschlussentwurf online am 7.12.2020, kurz vor Inkrafttreten der Coronavirus-Impfverordnung (CoronaImpfV) am 18.12.2020 und dem Impfbeginn am 27.12.2020.

**Prof. Dr. Dieter Cassel** ist Emeritus für Wirtschaftspolitik und Gesundheitsökonomie an der Universität Duisburg-Essen, Campus Duisburg.

**Prof. Dr. Volker Ulrich** ist Ordinarius für Volkswirtschaftslehre, insbesondere Finanzwissenschaft, an der Universität Bayreuth.

Absehbar war allerdings die wegen anfänglich unzureichender Produktionskapazitäten weltweit extreme Knappheit der drei zuerst zugelassenen Impfstoffe von BioNTech/Pfizer, Moderna und AstraZeneca/Oxford. Dies hätte erfordert, den Kreis der zu priorisierenden Impfberechtigten darauf zahlenmäßig abzustellen und ihre Impfung nach Verfügbarkeit der Vakzine zeitlich zu takten. Stattdessen ist das BMG der Empfehlung der STIKO gefolgt und hat nicht nur mit etwa 40 Mio. Personen fast die Hälfte der erwachsenen Bevölkerung priorisiert, sondern auch in der ersten, erwartungsgemäß besonders kritischen Phase über 8,6 Mio. Personen mit höchster Impfpriorität aufgerufen, darunter etwa 5,4 Mio. über 80-Jährige (Cassel und Ulrich, 2021a, 14 ff.). Die Folge war eine mitten im zweiten Lockdown mit Lieferschwierigkeiten, Informationspannen und Terminvergabeproblemen nur ruckelnd angelaufene Impfkampagne, was dem Vertrauen in das Krisenmanagement von Bund und Ländern nachhaltig geschadet haben dürfte.

## Das AstraZeneca-Debakel

Ende 2020 wurde der Vektor-Impfstoff von AstraZeneca (AZ) von der European Medicines Agency (EMA) unbeschränkt für alle Altersgruppen über 18 Jahre zugelassen. Er gilt als sicher und vermeidet nahezu alle Hospitalisierungen mit schweren Krankheitsverläufen, weist aber im Vergleich zu den mRNA-Vakzinen von BioNTech und Moderna eine geringere Wirksamkeit auf (mindestens 63 % gegenüber 95 % bzw. 94 %). Auch konnte sie wegen einer zu geringen Datenbasis für unter 18- und über 64-Jährige zunächst noch nicht sicher belegt werden. Während die meisten EU-Länder und Großbritannien den AZ-Impfstoff bei allen über 18-Jährigen anwenden, ist er in anderen Ländern – wie Schweiz, USA und Südafrika – aus verschiedenen Gründen (noch) nicht zugelassen. Das BMG

hat auf Empfehlung der STIKO einen dritten Weg eingeschlagen, indem es den AZ-Impfstoff zunächst nur bei Erwachsenen im Alter von 18 bis 64 Jahren empfahl.

Dadurch wurden die bisherigen Priorisierungskriterien „Krankheitsverlauf“ und „Exposition“ um die Dimension „Impfstoffqualität“ erweitert.[2] Die daraufhin erforderliche Revision der Impfpriorisierung trat mit einer entsprechend geänderten CoronaImpfV am 8. Februar 2021 in Kraft. Sie teilte die vorrangig Impfberechtigten in zwei zu behandelnde Gruppen: In der einen durften nur die beiden mRNA-Vakzine appliziert werden, in der anderen der Vektor-Impfstoff von AZ. Nachdem die STIKO den letzteren lediglich eingeschränkt zur Impfung empfohlen und ihn damit in den Augen vieler – sicherlich unbeabsichtigt[3] – als minderwertig diskreditiert hatte, musste sich eine solche produktbezogene Segmentierung negativ auf die Impfbereitschaft der Betroffenen auswirken. Dies umso mehr, als die Impfstoffzuordnung in den bisher mit höchster oder hoher Priorität zu impfenden Personengruppen (§§ 2-3 CoronaImpfV) kaum nachvollziehbar war und damit einer Büchse der Pandora gleichkam (Cassel und Ulrich, 2021b, 3 f.).

Seitdem wurde beklagt, dass immer mehr Priorisierte eine Impfung mit dem AZ-Vakzin ablehnen und lieber ungeimpft bleiben oder bis zur breiten Verfügbarkeit der mRNA-Vakzine bzw. der Lieferbarkeit des erst am 11. März EU-weit zugelassenen Impfstoffs von Janssen-Cilag/Johnson&Johnson (J&J) voraussichtlich im zweiten Quartal 2021 warten wollen. Dieser Vektor-Impfstoff ist zwar ebenfalls nicht so wirksam wie die mRNA-Vakzine, bedarf aber nur einer Impfung und ist bei Kühlschranktemperatur transport- und lagerfähig. Es bleibt abzuwarten, wie sich die Präferenzen der noch nicht Geimpften bei den beiden Vektor-Impfstoffen, insbesondere nach dem zwischenzeitlichen Impfstopp von AZ, entwickeln. Immerhin lagen nach Angaben des RKI Mitte März noch 1,6 Mio. AZ-Dosen bzw. 47 % der Lieferungen bei den Impfzentren auf Halde. Dies verlangsamte die Impfkampagne und vergrößert später die noch zu erwartenden Impfengpässe ebenso, wie das aus Sicherheitsgründen in den meisten Bundesländern praktizierte Zurückhalten der Dosen für die zweite mRNA-Impfung. Dass es sich hierbei nicht um vernachlässigbare Größenordnungen handelt, zeigt die RKI-Statistik: Unmittelbar vor dem AZ-Impfstopp am 15. März 2021 waren von den bis dahin gelieferten 12,9 Mio. Dosen erst 9,7 Mio. (75,1 %) verimpft, darunter BioNTech zu 84 % und AstraZeneca zu 53 %.[4]

Die Lage hat sich weiter zugespitzt, nachdem das BMG auf Anraten des Paul-Ehrlich-Instituts (PEI) die Verimpfung des AZ-Vakzins überraschend ausgesetzt hatte und die Zentren Tausende wartende Impflinge ungeimpft nach Hause schicken mussten – nicht wenige davon trotz ihrer Bereitschaft, sich auf eigenes Risiko mit dem AZ-Stoff impfen zu lassen. Anlass dazu waren hierzulande sieben Fälle von höchst seltenen, bei 20 % der Patienten letal verlaufenden Hirn- bzw. Sinusvenenthrombosen mit drei Toten. Sie traten wie schon zuvor in einigen anderen Ländern – darunter in Frankreich, Italien und Spanien – jeweils in zeitlich geringem Abstand zur Impfung auf. Bezogen auf die bis dahin 1,6 Mio. verabreichten AZ-Dosen lag die Wahrscheinlichkeit, zeitlich versetzt zur Impfung diese Krankheit zu erleiden, bei nur 0,000004 (1:250.000) bzw. daran zu sterben bei 0,000002 (1:500.000). Demnach wäre das Risiko, an COVID-19 zu sterben, deutlich höher als an der vermuteten, bis dato noch nicht bestätigten Thrombosewirkung des AZ-Vakzins.

Es sei dahingestellt, ob angesichts dessen die Aussetzung der Impfungen mit einem ansonsten unbestritten sicheren und wirksamen Vakzin als „reine Vorsichtsmaßnahme“ (Spahn) bei noch ruckelnder Impfkampagne inmitten der dritten Infektionswelle verhältnismäßig war.[5] Jedenfalls dürften das Vertrauen in das Impfmanagement und die Akzeptanz des AZ-Vakzins gelitten und sich die Impfbereitschaft zumindest temporär verringert haben – an die dadurch Infizierten, Erkrankten und Gestorbenen gar nicht zu denken.

2 Die STIKO nahm die AZ-Zulassung zum Anlass, ihre bisherige weitgehend „personalisierte Priorisierung“ der Impfberechtigten nach Alter, Vorerkrankungen und Exposition nun auch nach Maßgabe der Impfstoffeigenschaften zu revidieren und dem BMG als neue „Feingranulierung“ (STIKO) zu empfehlen. Aufgrund neuester Studiendaten wurde allerdings von Bund und Ländern erwartet, dass sie kurzfristig die Altersbegrenzung der AZ-Anwendung aufhebt (BKMPK, 2021, 4). Dem ist die STIKO schon am nächsten Tag nachgekommen.

3 Der STIKO-Vorsitzende Mertens gesteht inzwischen ein: „Das Ganze ist einfach irgendwie schlecht gelaufen“ (FAZ vom 1. März 2021). Das gilt auch für den Ratschlag von Minister Spahn, man solle sich zunächst mit AZ impfen und mit mRNA nachimpfen lassen, sobald genügend Dosen für alle da seien (ZDFheute vom 20. Februar 2021).

4 Bei den noch nicht verbrauchten BioNTech-Dosen dürfte es sich überwiegend um angebotsbedingte Sicherheitsreserven für die zweite Impfung handeln, die umso geringer sein können, je verlässlicher die Lieferzusagen sind und je größer das Intervall zwischen erster und zweiter Impfung sein kann. Angesichts dessen hatte das BMG schon frühzeitig empfohlen, beim AZ-Stoff keine Rücklage zu bilden, sodass seine hohen Bestände im Wesentlichen nachfrageseitig mit einer produktspezifischen Impfzurückhaltung der Priorisierten und einer anfänglich zu starr gehandhabten Priorisierung erklärbar ist.

5 Dies bedarf der Diskussion: Schließlich sind Rolle und Verantwortung von PEI und STIKO als wissenschaftliche Berater und BMG als politischer Entscheider hinsichtlich des angerichteten Schadens grundsätzlich zu klären. Das dürfte auch für das Selbstbestimmungsrecht der Impfwilligen gelten, sich auf eigenes Risiko mit einem zugelassenen Stoff impfen zu lassen, solange keine Impfpflicht besteht (Kliemt, 2021; Niemeyer, 2021). Auch ist erklärungsbedürftig, warum der AZ-Impfstoff schon nach drei Tagen wieder zur unbeschränkten Anwendung freigegeben wurde, obwohl während des Impfstopps keine neuen Erkenntnisse zu gewinnen waren – mit Ausnahme der Notwendigkeit einer besseren Aufklärung über die Nebenwirkungsrisiken.

### Es wird geimpft – und keiner geht hin

Bereitstehende Impfstoffe bei neuerlich anrollender Infektionswelle und gelockerten Kontaktbeschränkungen nicht zu verimpfen, ist ein GAU der Pandemiebekämpfung – entstehen doch damit vermeidbare Schäden durch nicht verhinderte Infektionen, Verlängerung oder Verschärfung des Lockdowns sowie Vertrauensverlust mit Verringerung der Impfbereitschaft. Deshalb haben sich Bund und Länder auf ihrer Konferenz der Bundeskanzlerin mit den Ministerpräsident:innen vom 3. März 2021 darauf geeinigt, die für die Zweitimpfung zurückgehaltenen Dosen deutlich zu reduzieren und die zulässigen Intervalle zwischen erster und zweiter Impfung möglichst auszuschöpfen (BKMPK, 2021, 3 ff.). Auch können nun Personen der zweiten Priorisierungsgruppe produktbezogen „im fließenden Übergang" geimpft werden, obwohl die am höchsten Priorisierten noch nicht komplett immunisiert sind. Damit legitimierte man das Vorziehen nachrangig Priorisierter beim AZ-Vakzin, das von den Ländern schon längst praktiziert wurde. Schließlich sollen spätestens ab April auch Haus-, Fach- und Betriebsärzte mit Impferfahrung unter Beachtung der Priorisierung einbezogen werden, um das Impftempo merklich beschleunigen zu können. Diese Beschlüsse – wie insbesondere auch die zur Höherstufung der Beschäftigten in der Kinder- und Grundschulbetreuung – waren längst fällig, reichen aber bei Weitem nicht für eine erfolgversprechende Impfstrategie aus (Cassel et al., 2020, 66 ff.).

Primäres Ziel der Nationalen Impfstrategie des BMG ist der individuelle Impfschutz (Personenschutz) zur Vermeidung von Hospitalisierung und schweren Krankheitsverläufen, was sich in der CoronaImpfV als „personalisierte Priorisierung" konkretisiert. Außerdem soll die Corona-Pandemie mit einer raschen Durchimpfung der Bevölkerung zum Erreichen der Herdenimmunität (Bevölkerungsschutz) bekämpft werden. Dazu bedarf es aber einer „institutionalisierten Priorisierung" zur gezielten Immunisierung potenzieller Superspreader, Bekämpfung von Hotspots und Unterbrechung von Infektionsketten einerseits sowie eines effektiven Impfmanagements bei hoher Impfbereitschaft zur Vermeidung von Engpässen der Durchimpfung andererseits (Cassel und Ulrich, 2021a, 4 ff.).

Die bis dato ruckelige Impfkampagne ist weitgehend dem knappen Impfstoffangebot bis Ende Februar geschuldet. Seitdem steigt die Zahl der täglichen Impfungen infolge des verbesserten Impfstoffangebots tendenziell deutlich an, hat aber erst am 10. März 2021 die 250.000-Marke überschritten und durch den AZ-Impfstopp vorübergehend einen merklichen Rückschlag erlitten. Da mit dem Vektor-Impfstoff von J&J erst kürzlich ein weiteres Vakzin zugelassen wurde und der Impfstoff des russischen Gamaleja-Instituts (Sputnik V) vor der EU-Zulassung steht sowie die Produktionslinien der bereits zugelassenen Produkte immer noch erweitert werden, ist absehbar, wann die stationären und mobilen Impfkapazitäten der Impfzentren nicht mehr ausreichen werden, um die gelieferten Dosen zu verbrauchen: Nach der Impfstoffknappheit droht also ein Mangel an Impfkapazitäten. Schließlich ist irgendwann auch die Zahl der Geimpften so weit gestiegen, dass sich immer weniger Impfwillige finden lassen und die nicht vorhandene Impfbereitschaft zum Engpass beim Erreichen der Herdenimmunität werden könnte. Vor diesem Hintergrund ist fraglich, wie gut die Politik auf die absehbaren Problemlagen vorbereitet ist.

### Impfstoffe, Impfkapazitäten und Impfbereitschaft

Anfang Februar hat das BMG erstmals quantitative Modellberechnungen für eine verbesserte Zielgenauigkeit und Planbarkeit der Impfstoffversorgung vorgelegt (BMG, 2021a). Damit soll gewährleistet werden, dass die Länder das gemeinsame Ziel, die gelieferten Impfstoffe schnellstmöglich zu verwenden, tatsächlich erreichen. Grundlage dafür sind fest zugesagte bzw. geschätzte Liefermengen und Lieferzeitpunkte der schon angewendeten oder bis Ende 2021 erwartungsgemäß noch zugelassenen Impfstoffe. Während BioNTech und Moderna bis Ende März erst 13,0 Mio. Dosen liefern können, soll der Lieferumfang bis Ende 2021 insgesamt 178,4 Mio. Dosen betragen, womit 89,2 Mio. Menschen immunisiert werden könnten. Hinzu kommen noch das AZ-Kontingent mit insgesamt 56,3 Mio. Dosen[6] und die auf zusammen 88,8 Mio. Dosen geschätzten Lieferungen der erwartungsgemäß im zweiten Quartal zugelassenen Impfstoffe von J&J und CureVac bzw. von Sanofi/GlaxoSmithKline (GSK) im vierten Quartal. Bis Ende 2020 sind also insgesamt 323,5 Mio. Impfdosen zu erwarten. Dabei braucht das J&J-Vakzin nur einmal verimpft zu werden, sodass mit seinen zugesagten 36,7 Mio. Dosen die entsprechende Zahl von Personen immunisiert werden könnte.

Selbst wenn es zu Lieferausfällen käme, würden genügend Impfstoffe für alle 83,1 Mio. Menschen in Deutschland da sein. Das klingt hoffnungsvoll und gibt Anlass darüber nachzudenken, was mit den 200 Mio. bestellten, aber nicht benötigten Dosen geschehen soll – jetzt schon leer auf Termin verkaufen oder lieber verschenken? Vielleicht werden aber noch weitere Booster-Impfungen nötig, um die Bevölkerungsimmunität dauerhaft zu erhalten. Dann könnten sich die überschüssigen Dosen – ihre Haltbar-

6 Schon vor dem temporären Impfstopp hatte AstraZeneca das EU-Lieferkontingent auf die Hälfte gekürzt, könnte nun aber wegen eines möglichen weltweiten Nachfragerückgangs seine Lieferverpflichtungen doch noch erfüllen.

keit vorausgesetzt – sogar als segensreich erweisen. Die Aussicht auf genügend Impfstoff täuscht jedoch über zwei Probleme hinweg: Die in den ersten drei Quartalen von 18,3 über 77,1 auf 126,6 Mio. Dosen explosionsartig zunehmenden Lieferungen müssen zeitnah auch verabreicht werden. Das erfordert angebotsseitig hinreichende Impfkapazitäten und nachfrageseitig genügend Impfwillige.

### Impfkapazitäten von Arztpraxen

Um das Impfmanagement hierauf einstellen zu können, hat das BMK in Zusammenarbeit mit dem Zentralinstitut für die Kassenärztliche Versorgung in Deutschland (Zi, 2021a) ein Online-Tool entwickelt, mit dem Bund und Länder verschiedene Impfszenarien für den zeitlichen und quantitativen Ablauf der Impfkampagne berechnen und die Auswirkungen alternativer Vorgehensweisen simulieren zu können. Dies ermöglicht es, planerisch so wichtige Fragen wie die nach den wöchentlich verfügbaren Impfstoffmengen und der dafür benötigten Impfkapazität in den Impfzentren und Arztpraxen zu beantworten. Auch lässt sich der bevölkerungsbezogene Impfschutz abschätzen, der mit unterschiedlichen Impfstoffmengen, Impfkapazitäten und Verimpfungsintervallen erreichbar ist (Zi, 2021b). Die Modellierungen ergeben, dass die Phase der Impfstoffknappheit im April 2021 zu Ende geht und stattdessen eine Phase knapper Impfkapazitäten droht, sofern die 433 Impfzentren ihre angebliche, aber bisher noch keineswegs ausgelastete Impfkapazität von 340.000 Impfungen pro Tag nicht rasch auf über 550.000 aufstocken und die Vertragsärzt:innen arbeitstäglich nicht mindestens noch 1 Mio. Dosen zusätzlich applizieren. Um Impfstaus zu vermeiden, müssten also wöchentlich 9 Mio. Dosen verimpft werden,[7] sodass die gesamte erwachsene Bevölkerung in etwa 14 Wochen immunisiert wäre und rechtzeitig zu Beginn des Sommerurlaubs zur Normalität zurückkehren könnte.

Es bleibt abzuwarten, ob sich ein derart hohes Impfpotenzial wie geplant mobilisieren lässt. Entscheidend ist jedoch, dass das BMG seine Impfstrategie nach langem Zögern auf die veränderte Impfstoffverfügbarkeit eingestellt hat und nun die termingerechte Einbeziehung der niedergelassenen Ärzt:innen vorbereitet. Hierzu haben verschiedene Faktoren beigetragen: zum einen die Verfügbarkeit von praxistauglichen Vektorimpfstoffen (AZ und demnächst J&J), zum anderen der bevorstehende Abschluss der Impfungen mit höchster Priorität und eine flexiblere Handhabung auf den noch folgenden Prioritätsstufen sowie nicht zuletzt der immer stärkere Druck aus Ärzteschaft und Öffentlichkeit. Was in der Hektik der vollzogenen Strategiewende aus dem Blick geraten zu sein scheint, ist die möglicherweise zu geringe Impfbereitschaft als nächste Hürde auf dem Weg zur Herdenimmunität. Dieses Problem soll anhand der Abbildung 1 verdeutlicht werden.

### Mangelnde Impfbereitschaft

Darin veranschaulicht die Kurve mit Zahlenangaben den monatlich kumulierten prozentualen Anteil der Bevölkerung, der mit den voraussichtlich bis Ende 2021 verfügbaren Dosen aller sechs Impfstoffe immunisiert sein könnte.[8] Sie zeigt, dass 100 % der Bevölkerung bereits Ende Juli mit den bis dahin zu erwartenden Impfstofflieferungen durchgeimpft sein könnten. Ob dies gelingt, hängt jedoch von den verfügbaren Impfkapazitäten ab. In einem ersten Szenario werden für die Impfzentren (rautierte Kurve) nach bisherigen Schätzungen 200.000 Impfungen pro Tag im ersten Quartal und 300.000 in den folgenden Quartalen unterstellt.[9] Im Vergleich der beiden Kurvenverläufe zeigt sich die bereits hinter uns liegende Impfstoffknappheit, bei der weit weniger Dosen geliefert wurden als hätten verimpft werden können (Phase-I-Knappheit). Ab April würde jedoch das Gegenteil eintreten (Phase-II-Knappheit), wenn nicht die Impfkapazität der Zentren massiv aufgestockt oder niedergelassene Impfärzte einbezogen werden könnten. Dies zeigt das zweite Szenario, bei dem zusätzlich die von der KBV in Aussicht gestellten 5 Mio. Impfungen pro Woche unterlegt sind (gepunktete Kurve).

Nimmt man die in Abbildung 1 als Waagerechte eingezeichnete Immunisierung von 80 % der Bevölkerung als Zielmarke der Impfkampagne, wird die strategische Be-

7 Die Kassenärztliche Bundesvereinigung (KBV) rechnet damit, dass 50.000 der bundesweit 75.000 Arztpraxen arbeitstäglich 20 Dosen verimpfen könnten (KBV und Zi, 2021, 1), was einer Kapazität der niedergelassenen Ärzteschaft von 1 Mio. Impfungen pro Arbeitstag bzw. 5 Mio. pro Woche entspräche. Darin sind Betriebs-, Krankenhaus-, Zahn- und notfalls Tierärzt:innen noch nicht eingeschlossen, sodass auch die von Bundeskanzlerin Merkel und Vizekanzler Scholz in Interviews genannte Impfkapazität von 9,5 bzw. 10 Mio. Impfungen pro Woche nicht gänzlich unrealistisch erscheinen. Die ab 5. März 2021 den 50.000 Arztpraxen wöchentlich zugeteilten 20 Dosen, zusammen 1 Mio. Dosen pro Woche, sind allenfalls das Starterkit dafür.

8 Mit den bis Ende 2021 vereinbarten Lieferungen könnten (vgl. Abbildung 1) rein rechnerisch sogar 217 % der Bevölkerung immunisiert werden. Sie beruhen auf quartalsweisen Prognosedaten des BMG (2021a; Update vom 22. Februar 2021). Linear auf die Monate verteilt und dann kumuliert, wurden sie auf die maximale Zahl von Geimpften mit Vollschutz umgerechnet. Dabei wurde berücksichtigt, dass der J&J-Impfstoff nur einmal verabreicht wird. Dagegen bleiben die unterschiedlichen produktspezifischen Intervalle zwischen den Impfungen aus Gründen der Vereinfachung unberücksichtigt, obwohl sie leicht verzögernd wirken.

9 Bei den Impfkapazitäten wurden die Zi-Modellierungen (Zi, 2021a) als Untergrenze und die KBV-Schätzungen (KBV und Zi, 2021) als Obergrenze angenommen.

Abbildung 1
**Phasen einer Impfkampagne mit allen 2021 verfügbaren Impfstoffen**

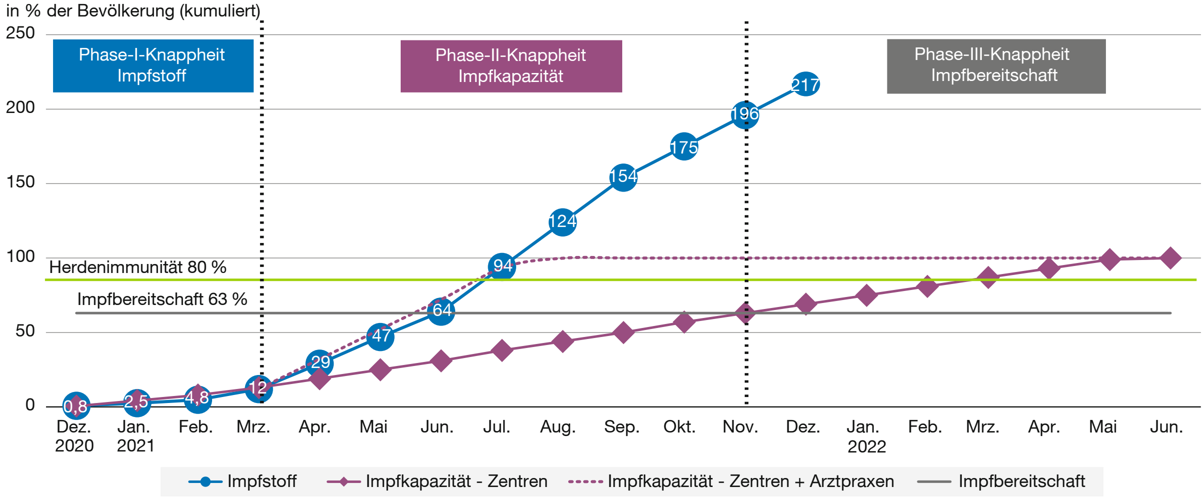

Quelle: eigene Darstellung und Berechnung nach BMG-Daten vom 22. Februar 2021 (BMG, 2021b).

deutung der Impfkapazität deutlich:[10] Bliebe es bei den im ersten Szenario unterstellten maximal 300.000 Impfungen pro Tag bzw. 2,1 Mio. pro Woche, würde sie erst im März 2022 erreicht werden. Im zweiten Szenario mit annahmegemäß 7,1 Mio. Impfungen pro Woche wäre man schon im Juli 2021, also neun Monate früher, am Ziel – vorausgesetzt, es ist genügend Impfstoff vorhanden.[11]

Allerdings müsste dann die Impfbereitschaft mindestens bei 80 % der Gesamtbevölkerung bzw. 95 % der Erwachsenen liegen, solange unter 18-Jährige für Impfungen nicht in Betracht kommen. Wie die zweite Waagerechte zeigt, liegt die Impfbereitschaft aktuell bei 63 % bezogen auf die Gesamtbevölkerung.[12] Würde sich daran nichts ändern, wäre die Impfkampagne im ersten Szenario bereits im November 2021 aus Mangel an Impfwilligen zu Ende (Phase-III-Knappheit; rautierte Kurve) – und im zweiten (gepunktete Kurve) sogar schon im Juni; denn bis zur Herdenimmunität fehlten dann jeweils noch 17 % Geimpfte in der Bevölkerung.

## Mit BioNTech und Moderna allein ans Ziel

Schon die Impfbeschränkung für über 64-Jährige, die Anfang Januar für das AZ-Vakzin verfügt und auf Drängen der BKMPK Anfang März wieder aufgehoben wurde, hat bei den unter 65-Jährigen verbreitet zu einer produktbezogene Impfverweigerung geführt (Cassel und Ulrich, 2021b). Es ist nicht ausgeschlossen, dass der Impfstopp das Vertrauen in das AZ-Produkt nochmals beschädigt und auch generell die Impfbereitschaft beeinträchtigt hat. Deshalb wäre es gut zu wissen, ob man den Vertrauensbonus der mRNA-Vakzine nutzen und die Herdenimmunität gegebenenfalls auch mit den von BioNTech und Moderna zugesagten Dosen allein erreichen könnte.

Hierfür haben wir analoge Berechnungen unter der Annahme angestellt, dass nur diese beiden Impfstoffe verwendet werden. Wie Abbildung 2 zeigt, bleibt es im ersten Szenario (rautierte Kurve) beim Ende der Impfkampagne im November 2021 wegen mangelnder Impfbereitschaft (Phase-III-Knappheit). Dagegen sind im zweiten Szenario (gepunktete Kurve) die Impfkapazitäten deutlich höher als die Liefermengen. Dadurch kann der Bevölkerungsteil der Geimpften nur der Impfstoffverfügbarkeit entsprechend bis zur Grenze der Impfbereitschaft zunehmen, die im August 2021 erreicht wäre – also nur zwei Monate später als in Abbildung 1. Aber selbst bei ausreichender Impfbereitschaft würde sich das Erreichen der Herdenimmunität nur um gut zwei Monate verzögern. Von daher ließe sich bei

10 Anfangs wurde bei SARS-CoV-2 die Herdenimmunität für die mRNA-Vakzine mit 65 % bis 70 % Geimpften in der Gesamtbevölkerung angesetzt; aufgrund des Vordringens der weit infektiöseren Mutanten einerseits und der zunehmenden Verimpfung von Vektor-Impfstoffen mit geringerer Wirkung andererseits geht man aktuell von 80 % aus. Moore et al. (2021) kommen neuerdings für UK zu dem Ergebnis, dass selbst diese Zahl noch viel zu gering sein dürfte.

11 Im Ergebnis ähnlich eine Studie der Hans-Böckler-Stiftung (IMK, 2021).

12 Nach einer aktuellen Befragung von Bitkom Research möchten sich 72 % der Bevölkerung über 16 Jahren impfen lassen. Das sind gut 52 Mio. Menschen bzw. 63 % der Gesamtbevölkerung (Berg, 2021, 2). Das Hamburg Center for Health Economics (HCHE; J. Schreyögg) ermittelte in einer EU-weiten Routinebefragung eine im Januar etwas gestiegene Impfbereitschaft von 62 % für Deutschland, das damit aber immer noch den vorletzten Platz einnimmt (HCHE, 2021).

Abbildung 2

**Impfkampagne mit den 2021 verfügbaren mRNA-Impfstoffen von BioNTech und Moderna**

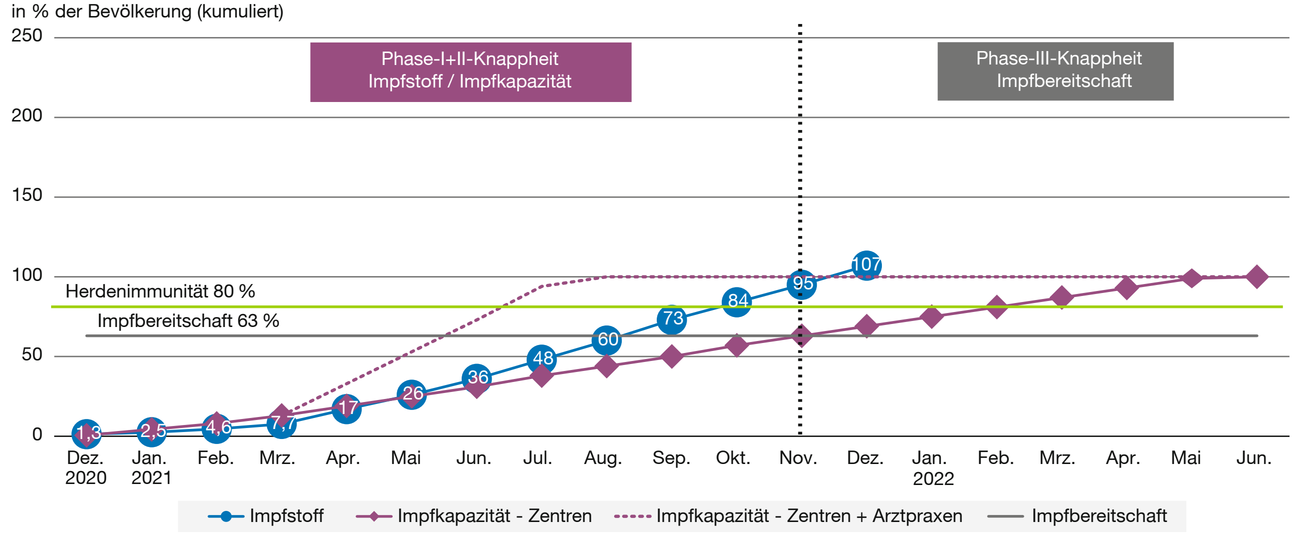

Quelle: eigene Darstellung und Berechnung nach BMG-Daten vom 22. Februar 2021 (BMG, 2021b).

ausreichenden Impfkapazitäten die Impfkampagne notfalls auch mit den beiden mRNA-Vakzinen in kurzer Zeit bis Ende September 2021 erfolgreich abschließen – sofern nur genügend Impfwillige da sind.

Diese Projektionen bzw. Status-quo-Prognosen sind jedoch nicht als realistische Vorhersagen künftiger Ereignisse zu verstehen. Vielmehr sollen sie eine Vorstellung davon geben, wie sich die Dinge unter bestimmten, gegebenenfalls alternativen Annahmen bzw. Szenarien entwickeln und durch Eingriffe gesteuert werden könnten. So lassen die beiden Abbildungen erkennen, dass die Impfkampagne drei Phasen der Knappheit durchlaufen kann, von denen die erste mit großer Wahrscheinlichkeit bereits beendet ist, die zweite gerade begonnen hat und die dritte voraussichtlich im Sommer bevorsteht. Dies setzt allerdings voraus, dass die Impfkapazität durch Einbeziehung der niedergelassenen Ärzteschaft auf etwa 7 Mio. Impfungen pro Woche erhöht und von den 44 Mio. bis dahin noch nicht vakzinierten Impfwilligen auch genutzt wird.[13] Bis zur Herdenimmunität fehlen dann immer noch 14 Mio. (17 %) Impfwillige, die aus dem Potenzial der etwa 30 Mio. (37 %) Impfverweigerer oder noch Unentschlossenen zu rekrutieren wären.[14]

13 Bis dato sind erst 7,5 Mio. Impfberechtigte mindestens einmal geimpft worden, sodass von den 52 Mio. (63 %) Impfbereiten noch 44,5 Mio. zur Impfung anstehen, die sich in etwa auf 29,5 Mio. Priorisierte und 15 Mio. Nicht-Priorisierte aufteilen. Schließlich gibt es auch unter den Priorisierten – erfahrungsgemäß gerade unter dem medizinisch-pflegerischen Personal – nicht wenige Impfverweigerer.

14 Die Lage würde sich schlagartig entspannen, wenn auch die etwa 13 Mio. unter 16-Jährigen geimpft werden könnten.

## Schnell und unbürokratisch impfen

Soll die Impfkampagne nicht an Querdenkern, Impfgegnern und Unbesorgten scheitern, muss die Impfstrategie auch für die dritte Phase gerüstet sein. Hierauf haben schon seit längerem STIKO, Deutscher Ethikrat, Leopoldina und andere hingewiesen und vor allem vertrauensbildende Maßnahmen wie Informationen, Aufklärung, Appelle usw. gefordert. Sie allein werden aber nur schwerlich Erfolg haben, hat die bisherige Impfkampagne doch schon mehr Vertrauen zerstört als aufgebaut. Aus ökonomischer Sicht dürfte eine steigende Impfbereitschaft ohnehin eher mit Anreizen und Sanktionen („Carrot and Stick“) erreichbar sein. Dies lässt sich aus den externen Effekten des Impfens und Nichtimpfens ableiten, die positiv („nützlich“) oder negativ („schädlich“) sein können.[15]

Geimpfte erhalten nämlich zum einen die positiven externen (Schutz-)Effekte, die sie zum Nutzen Dritter und letztlich für die Gesellschaft zum Erreichen der Herdenimmunität bewirken, nicht kompensiert; zum anderen verursachen Personen, die sich nicht impfen lassen, negative externe Effekte zum Schaden Dritter – insbesondere gegenüber Risikogruppen wie auch der Gesellschaft als Ganzes –, für den sie nicht aufkommen müssen. Außerdem profitieren sie als Trittbrettfahrer von sinkenden Ansteckungsrisiken durch Geimpfte. Dadurch wird Impfen zu einem Gut mit positiven externen Effekten, bei dem die

15 Siehe hierzu und im Folgenden Cassel et al. (2020, 59 ff.), Kliemt (2020), Wein (2021).

private Nachfrage hinter dem gesellschaftlich erwünschten Ausmaß zurückbleibt. Pragmatisch gesehen gilt also, die externen Effekte zu internalisieren und so die individuelle Impfbereitschaft mit kompensatorisch wirkenden Mitteln zu erhöhen.

- Der Carrot-and-Stick-Devise würden ökonomisch gesehen am ehesten finanzielle Anreize zum Impfen und Sanktionen des Nicht-Impfens entsprechen. Der Höhe nach wirksame Geldbeträge zur generellen Belohnung des Impfens zu zahlen, dürfte bei etwa 65 Mio. zu Impfenden fiskalisch rasch an Grenzen stoßen, im Wesentlichen aus Mitnahmeeffekten bestehen und politisch schwer vermittelbar sein. Und das Impfverweigern durch Strafzahlungen zu sanktionieren, um die Impfbereitschaft zu erhöhen, wird bei notorischen Impfgegnern kaum erfolgreich sein und juristisch noch vor dem Ende der Pandemie auch nicht durchsetzbar.

- Erfolgversprechender ist es, das Impfen kostenlos anzubieten, wie es in Deutschland und auch weltweit schon häufig geschieht. Darüber hinaus sollte es aber auch möglichst geringe monetäre und nicht monetäre (intangible) Nebenkosten – wie Informations-, Wege- und Zeitkosten oder Nebenwirkungen – verursachen. Von daher werden sich das steigende Impfstoffangebot und die zunehmenden Impferfahrungen ebenso positiv auswirken wie das demnächst schrittweise mögliche Impfen durch niedergelassene Impfärzt:innen.

- Besonders schädlich wirken sich die intangiblen Kosten der Terminvergabeprozeduren mit ihren Warteschlangen aus. Dies ist zu bedenken, wenn die Bundeskanzlerin nach abgeschlossener Durchimpfung der Priorisierten ihr versprochenes „Impfangebot an alle" einlöst; denn es wird sich dann mit einem Schlag an die verbliebenen 30 Mio. noch Ungeimpften richten. Selbst wenn niedergelassene Impfärzt:innen den Problemdruck verringern dürften, sollten jetzt schon die Vorkehrungen für elektronische Wartschlangen getroffen werden: Darin könnten sich die Impfwilligen bei Zentren und Arztpraxen virtuell einreihen und flexibel zur Impfung aufrufen lassen.

- Dazu würde auch eine flexiblere Handhabung der Priorisierung durch Vorziehen von Impfungen nach Verfügbarkeit einzelner Impfstoffe gehören, um Impfstaus zu vermeiden („Impfungen mit produktbezogenen Geschwindigkeiten"). Auch könnte sich die Priorisierung erübrigen, sobald die Impfnachfrage der Priorisierten unter das verfügbare Impfangebot fällt; denn je leichter der Zugang zur Impfung ist, umso höher wird die Impfbereitschaft sein.

- Auch sollten die Kosten verlässlicher Informationen über Qualität und Sicherheit der verimpften Stoffe, die Bedeutung der Impfpriorisierung, die persönlichen und sozialen Vorteile des Impfens und auch die Wahrscheinlichkeit von Risiken und Nebenwirkungen so gering wie möglich sein. Dies wäre regierungsseitig durch ein professionelles Entscheidungs- und Informationsmanagement und ärztlicherseits durch vertrauensbasierte persönliche Aufklärung und Beratung der Impfberechtigten in den Arztpraxen zu gewährleisten. Vor allem dürfte sich ein Debakel wie beim AZ-Vakzin nicht wiederholen.

- Die Impfbereitschaft ist erfahrungsgemäß umso höher, je größer der erwartete individuelle Nutzen aus der Impfung ist. Dieser hängt wesentlich davon ab, wie wirksam der Impfstoff ist, welche Nebenwirkungen er hat, wie gut das Virus nach Reproduktion und Inzidenz bereits kontrolliert ist, inwieweit das Testen fälschlicherweise als ein die Impfung ersetzender Infektionsschutz gesehen wird, ob es bereits effektive Behandlungsmöglichkeiten von Covid-19 gibt und nicht zuletzt, welches Risiko besteht, schwer daran zu erkranken. Diese Faktoren wirken teilweise gegenläufig auf die Impfbereitschaft und sind noch dazu schwer steuerbar.

- Dagegen gilt als wirksamster Anreiz zur Erhöhung der Impfbereitschaft ein amtlicher „elektronischer EU-Impfpass". Er wurde schon früh gefordert (Cassel et al., 2020, 68; Cassel und Ulrich, 2021a, 23), scheint aber erst jetzt politisch durchsetzbar zu sein, nachdem es Anzeichen gibt, dass die bisher zugelassenen Vakzine die Transmission ausschließen. Die Anreizwirkung setzt allerdings voraus, dass Träger:innen des Impfpasses weitgehend von Lockdown- bzw. Mobilitätsbeschränkungen verschont bleiben. Darin ist keine Privilegierung der Geimpften zu sehen, sondern vielmehr eine nach erfolgter Immunisierung mögliche Rückkehr zur Normalität, die ihnen zuvor durch pandemiebedingte Einschränkungen ihrer Grundrechte verwehrt war.

- Gleichsam als Ultima Ratio ließe sich die Zahl der Impflinge auch mit einer gesetzlichen Impfpflicht erhöhen. Dafür käme jedoch keine generelle Impfpflicht in Betracht, ist sie doch eine ziemlich rigorose Methode zur Internalisierung der externen Impfeffekte: Sie würde unter den derzeitig noch moderaten Pandemie-Bedingungen die verfassungsrechtlich garantierten Grundrechte unverhältnismäßig stark einschränken. Dagegen wäre eine selektive Impfpflicht für bestimmte Berufsgruppen – wie das medizinisch-pflegerische Personal oder die Belegschaften systemisch relevanter Betriebe – schon aus pandemischen Gründen notwendig sowie ethisch und rechtlich vertretbar.[16] Sie ließe sich im Kontext mangelnder Impfbereitschaft damit begründen, dass sie einen notwendigen Beitrag zur Abwendung der negativen Wirkungen auf Dritte und die Gesellschaft als Ganzes leistet, die von Ungeimpften ausgehen.

Hieran wird deutlich, dass „Corona-Impfstoffe im Überfluss" nicht nur die Lösung eines Problems sind, sondern auch neue Probleme aufwerfen: Nun gilt es zu verhindern, dass am Ende zu wenig Impfwillige da sind, um bei gegebenem Wirkungsgrad der Vakzine und erhöhter Infektiosität ihrer Mutanten die gewünschte Herdenimmunität erreichen zu können. Die Lösung dafür heißt „AIDA": Aufklären, Impfen, Dokumentieren – und dann Alltag!

16 Zur ebenfalls früh erhobenen, aber bislang politisch nicht aufgegriffenen Forderung nach einer selektiven Impfpflicht und deren Ausgestaltung siehe Cassel et al. (2020, 66 f.); Cassel und Ulrich (2021a, 23).

**Title:** ***Coronavirus Vaccines in Abundance — What Then?***

**JEL Classification:** *I10, I11, I18*

## References

[CR1] Berg, A. (2021), Digital gegen Corona — Präsentation des Präsidenten von Bitkom — Bundesverband Informationswirtschaft, Telekommunikation und neue Medien — vom 23. Februar 2021, https://www.bitkom.org/Bitkom/Publikationen/index.jsp (22. März 2021).

[CR2] BKMPK — Bundeskanzlerin mit den Regierungschefinnen und Regierungschefs der Länder (2021), Beschluss der Videoschaltkonferenz am 3. März 2021, https://www.bundesregierung.de/resource/blob/997532/1872054/66dba48b5b63d8817615d11edaaed849/2021-03-03-mpk-data.pdf?download=1 (22. März 2021).

[CR3] BMG — Bundesministerium für Gesundheit (2021a), Modellierung im Rahmen der Nationalen Impfstrategie zur Ministerpräsidenten-Konferenz (MPK) am 10. Februar 2021, Anlage 1: Status quo und Modellierung des BMG; Anlage 2: Modellierung von Impfszenarien des Zi, Stand: 9. Februar 2021; Update: 22. Februar 2021.

[CR4] BMG — Bundesministerium für Gesundheit (2021b), Informationen zur Impfstoffbeschaffung gegen COVID-19. Liefermengen/Lieferzeitpunkt/Zulassung, Stand: 22. Februar 2021.

[CR5] Cassel D, Heigl A, Jäcker A, Ulrich V (2020). Impfstoff für alle — doch wie soll das gehen? Probleme der Verfügbarkeit und Verteilung von Covid-19-Impfstoffen. RPG — Recht und Politik im Gesundheitswesen.

[CR6] Cassel D, Ulrich V (2021). Corona-Impfpriorisierung: personalisiert oder institutionalisiert? Genese und Probleme der Regelung des Impfstoffzugangs nach §§ 2 bis 4 Coronavirus-Impfverordnung. RPG — Recht und Politik im Gesundheitswesen.

[CR7] Cassel D, Ulrich V (2021). Neue Impfpriorisierung — eine Büchse der Pandora?. dfg — Dienst für Gesellschaftspolitik.

[CR8] HCHE — Hamburg Center for Health Economics (2021), HCHE COVID-19 study, https://www.hche.uni-hamburg.de/corona.html (29. März 2021).

[CR9] IMK — Institut für Makroökonomie und Konjunkturforschung der Hans-Böckler-Stiftung (2021), Covid19-Durchimpfung der Bevölkerung in Deutschland bis Juli 2021 ist möglich. Eine Projektion von S. Dullien und A. Watt, *IMK Policy Brief*, 102, März, https://www.boeckler.de/pdf/p_imk_pb_102_2021.pdf (22. März 2021).

[CR10] KBV und Zi (Kassenärztliche Bundesvereinigung und Zentralinstitut für die kassenärztliche Versorgung) (2021), Erwachsene Bevölkerung könnte bis Ende Juli 2021 geimpft sein, Gemeinsame Medieninformation, 24. Februar, https://www.zi.de/fileadmin/images/content/PMs/Gem_MI-Zi-KBV_Impfmodellierung_2021-02-24.pdf (22. März 2021).

[CR11] Kliemt, H. (2020), Impfen und impfen lassen, *Wirtschaftliche Freiheit*, 18. Dezember, https://www.wirtschaftlichefreiheit.de/wordpress/?p=28404#more-28404 (22. März 2021).

[CR12] Kliemt, H. (2021), Per aspera ad AstraZeneca?, *Wirtschaftliche Freiheit*, 16. März, https://www.wirtschaftlichefreiheit.de/wordpress/?p=28859#more-28859 (22. März 2021).

[CR13] Moore, S., E. M. Hill, M. J. Tildesley, L. Dyson und M. J. Keeling (2021), Vaccination and non-pharmaceutical interventions for COVID-19: a mathematical modelling study, *The Lancet Infectious Diseases 2021*, Online 18. März, https://www.sciencedirect.com/science/article/pii/S1473309921001432?via%3Dihub (22. März 2021). 10.1016/S1473-3099(21)00143-2 PMC7972312 33743847

[CR14] Niemeyer, F. (2021), Entscheidung gegen Astrazeneca. Das ist der falsche Weg, Herr Spahn!, *Kommentar in ntv*, 16. März, https://www.ntv.de.

[CR15] Wein T (2021). Ist eine Impfpflicht gegen das Coronavirus nötig?. Wirtschaftsdienst.

[CR16] Zi — Zentralinstitut für die kassenärztliche Versorgung (2021a), Modellierung von Impfszenarien, Stand: 23. Februar, https://www.zi.de (22. März 2021).

[CR17] Zi — Zentralinstitut für die kassenärztliche Versorgung (2021b), Simulation der COVID19-Impfkampagne. Ein Tool des Zi Data Science Lab, Stand: 25. Februar, https://www.zidatasciencelab.de/cov19vaccsim (22. März 2021).

